# A Hidden Threat: Hydatid Cyst Masked by Bacterial Infection

**DOI:** 10.7759/cureus.72045

**Published:** 2024-10-21

**Authors:** Snehal Pathak, Nayab Muneeruddin Shaik, Blessy Daniel, Mohamed Habieb

**Affiliations:** 1 Internal Medicine, Peterborough City Hospital, Peterborough, GBR; 2 Internal Medicine, Northwest Anglia NHS Foundation Trust, Peterborough, GBR; 3 Internal Medicine, Royal Devon University Healthcare NHS Foundation Trust, Exeter, GBR; 4 Gastroenterology, Peterborough City Hospital, Peterborough, GBR

**Keywords:** abdominal sepsis, cyst hydatid, echinococcus cyst, hydatid serology, liver abcess, liver abscess aspiration, parasitology, praziquantel, pyogenic liver abscesses, tropical fever

## Abstract

Hydatid cysts are parasitic infections that primarily affect the liver, but complications like secondary bacterial infections can obscure the clinical presentation. Though infections are common in general practice, hepatic abscesses, particularly those associated with hydatid cysts, are rare and often manifest with non-specific symptoms, leading to delayed diagnosis. Given the risk of serious complications such as rupture and sepsis, maintaining a high index of suspicion is critical, especially in patients with risk factors like a history of travel to endemic regions, liver disease, or excessive alcohol consumption. This case report highlights the clinical challenge posed by a hydatid cyst complicated by bacterial infection. The patient's non-specific complaints initially masked the true pathology, resulting in a delayed recognition of the hepatic abscess. Prompt recognition of this condition, despite its rare presentation, is crucial for timely intervention and improved patient outcomes.

## Introduction

A liver abscess is a buildup of thick fluid in the intra-abdominal cavity, which may include blood, dead cells, or microorganisms. These abscesses typically arise after infections in the liver, bile ducts, or other areas of the body. Liver abscesses are predominantly pyogenic, constituting 50-80% of cases, while amoebic abscesses represent the second most common cause, accounting for approximately 10-40%. In contrast, parasitic liver abscesses other than amoebic, are extremely rare. The distribution can vary depending on geographical location and local healthcare conditions [1].

Hydatid disease, also known as echinococcosis, is a lethal zoonotic, pathogenic, and parasitic disease caused by the larval stage of *Echinococcus granulosus* [2]. Infection with *Echinococcus granulosus* remains a major issue in several countries and regions, where livestock farming is prevalent, such as parts of the Middle East, Mediterranean, South America, and Africa [3]. However, due to increased global travel and migration, cases can be seen worldwide, including in non-endemic areas. The hydatid cyst is mainly found in the liver (around 50-70% of cases) and is asymptomatic in most cases, often discovered accidentally during routine investigations or as a complication with bacterial infection [2].

Most patients with hydatid cysts are asymptomatic unless there is a complication. Complications of hydatid cysts are rare but can be fatal. They include rupture into the biliary tract, peritoneal cavity, thoracic cavity, and gastrointestinal tract, resulting in secondary bacterial infection which can lead to liver abscess formation and bacteremia [4]. Because of the wide differential diagnosis for complicated cysts, the diagnosis can be missed. We report a 45-year-old man who presented to the emergency department on two occasions before he was diagnosed with an infected hydatid cyst.

## Case presentation

Medical history and demographics

A 45-year-old male presented to the emergency department with a four-week history of fever, rigors and nausea, and severe para-umbilical pain radiating to his right subcostal region. He also reported loss of appetite and a 10 kg weight loss over the past three weeks. He did not report vomiting or diarrhea. Six days prior to this presentation, he attended the emergency department with a three-week history of fever, reduced appetite, fatigue, and pain in his right flank radiating to his back. He had mild right renal angle tenderness and a raised C-reactive protein level (219 mg/L) at that stage. No imaging studies were conducted during this visit. Based on his clinical presentation and bedside examination findings, he was discharged with oral co-amoxiclav antibiotics and scheduled for outpatient urology follow-up to treat right-sided pyelonephritis. His symptoms began after returning from a holiday in Israel. He had also travelled to Majorca, Spain in the preceding three months. Although a detailed dietary history was not obtained, there was no direct contact with farm animals or any pets. His past medical history was pertinent for hypertension. His regular medications included taking candesartan with variable compliance. His social history was pertinent for alcohol consumption above recommended limits. He was an ex-smoker, having quit over 25 years prior to presentation. Clinical examination on this attendance was pertinent for tenderness over the right upper quadrant without any signs of peritonism. His respiratory examination was unremarkable, as was his cardiovascular examination. Blood pressure was 138/77 mmHg, and his oxygen saturation was 96% on room air.

Investigations

His repeat blood work was pertinent for persistently elevated CRP levels of 225 mg/L. His other bloodwork including full blood count, liver function tests, electrolytes, and serum creatinine levels were unremarkable, save a marginally low albumin level of 33 g/L (Table 1).

**Table 1:** Results of blood investigations done at the emergency department. Results confirm the presence of systemic infection.

Test	Result	Reference value
White blood cells	9.8	4.0-11.0 x 10^9^/L
Red blood cells	4.61	4.40-6.50 x 10^12^/L
Haemoglobin	130	130-180 g/L
Platelets	581	150-400 x 10^9^/L
Lymphocytes	1.8	1.4-4.8 x 10^9^/L
Monocytes	0.9	0.1-0.8 x 10^9^/L
Eosinophils	0.0	0.1-0.6 10^9^/L
Basophils	0.0	0-0.20 x 10^9/L
Neutrophils	6.9	1.8-7.7 x 10^9^/L
C-reactive protein	225	<5 mg/L
Estimated glomerular filtration rate	90	>60 ml/min
Albumin	33	35-50 g/L
Total bilirubin	9	<21 µmol/L
Alanine transaminase	59	<41 U/L
Alkaline phosphatase	105	30-130 U/L
Total protein	71	60-80 g/L
Sodium	132	133-146 mmol/L
Potassium	5.0	3.5-5.3 mmol/L
Creatinine	89	59-104 µmol/L
Chloride	100	95-108 mmol/L
Urea	3.7	2.5-7.8 mmol/L

**Figure 1:**
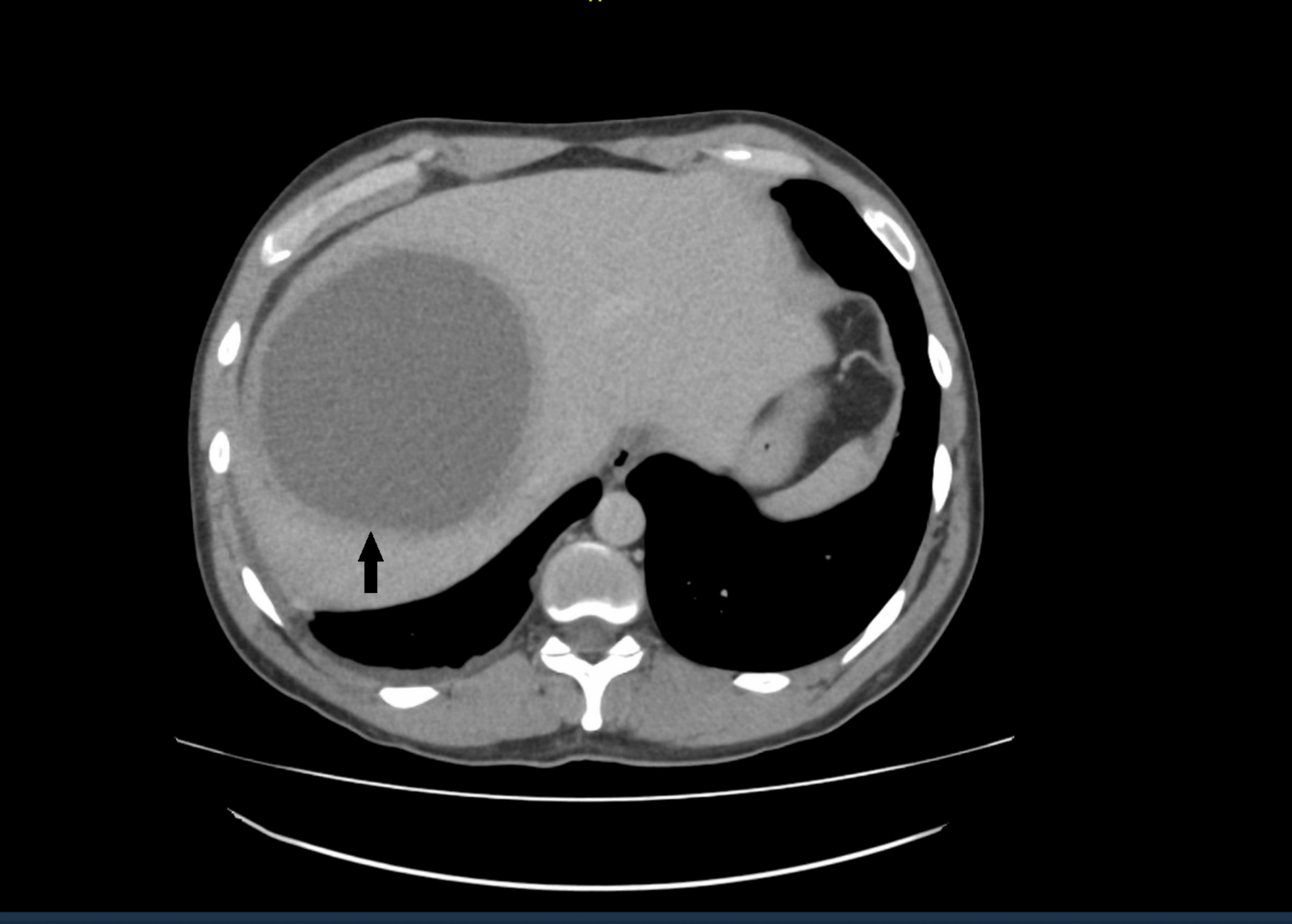
A computed tomography scan with contrast of the abdomen A cystic lesion in the right hepatic lobe with a thick-walled hepatic lesion measuring 12 cm x 11 cm can be seen exerting a local mass effect (black arrow).

**Figure 2:**
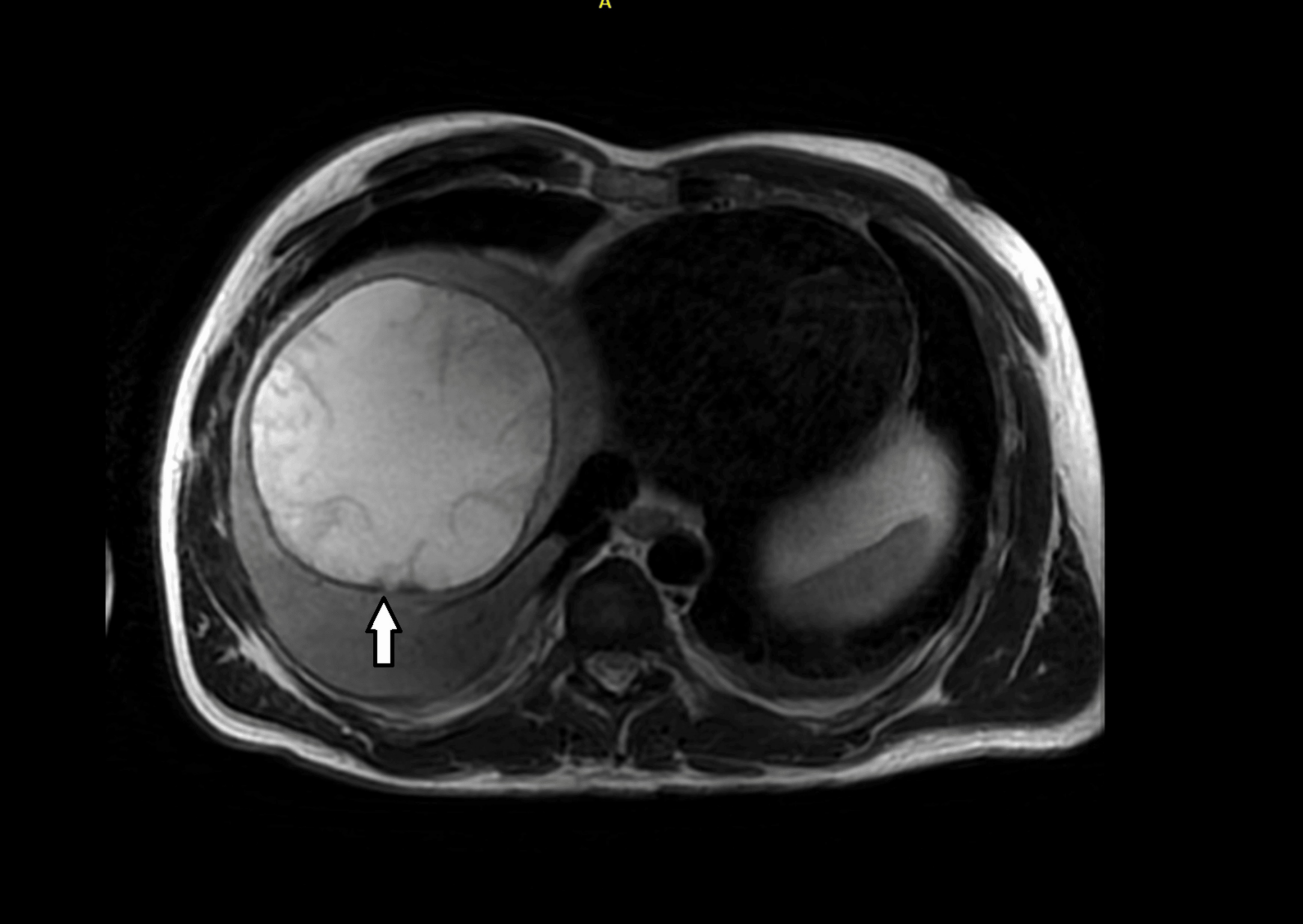
A magnetic resonance imaging scan with contrast of the abdomen A large solitary cystic liver lesion in the right lobe of the liver measuring 13 x 13 cm with thick and smooth wall enhancement; there are also some linear low-signal areas likely indicating septa, along with debris within the cyst (white arrow)

Given his clinical presentation, an urgent contrast-enhanced computed tomography scan of his abdomen was carried out, which showed a large solitary thick-walled cystic lesion in the right hepatic lobe with mass effect and splaying of the right and middle hepatic veins (Figure 1). A magnetic resonance imaging scan confirmed a large solitary cystic liver lesion in the right lobe, measuring 13 cm x 13 cm. There was no abnormal nodular soft tissue enhancement within the cyst. The cyst had a mass effect on the right hepatic veins, with no bile duct dilatation (Figure 2).

Given the current imaging findings and clinical history, the differential diagnosis at this stage includes a liver abscess resulting from bacterial, amoebic, or parasitic infections, as well as a hydatid cyst and a cystic neoplasm. These conditions warrant further evaluation to determine the underlying cause.

Treatment

He was initially started on intravenous co-amoxiclav and metronidazole but due to persistent spikes in temperature and unresolved clinical symptoms, this was switched to piperacillin tazobactam combination. Despite the ongoing treatment, the patient continued to experience intermittent fever spikes. Further tests including blood, stool, and urine cultures and stool for ova, cysts amoebic, parasites, and hydatid serology and microscopy were sent. His results were pertinent for a positive ELISA test for echinococcus, and negative for every other test for amoeba. After further consultation with the regional tropical disease team, praziquantel was started in place of albendazole, due to concerns that albendazole could weaken the cyst walls, increasing the risk of rupture.

**Figure 3:**
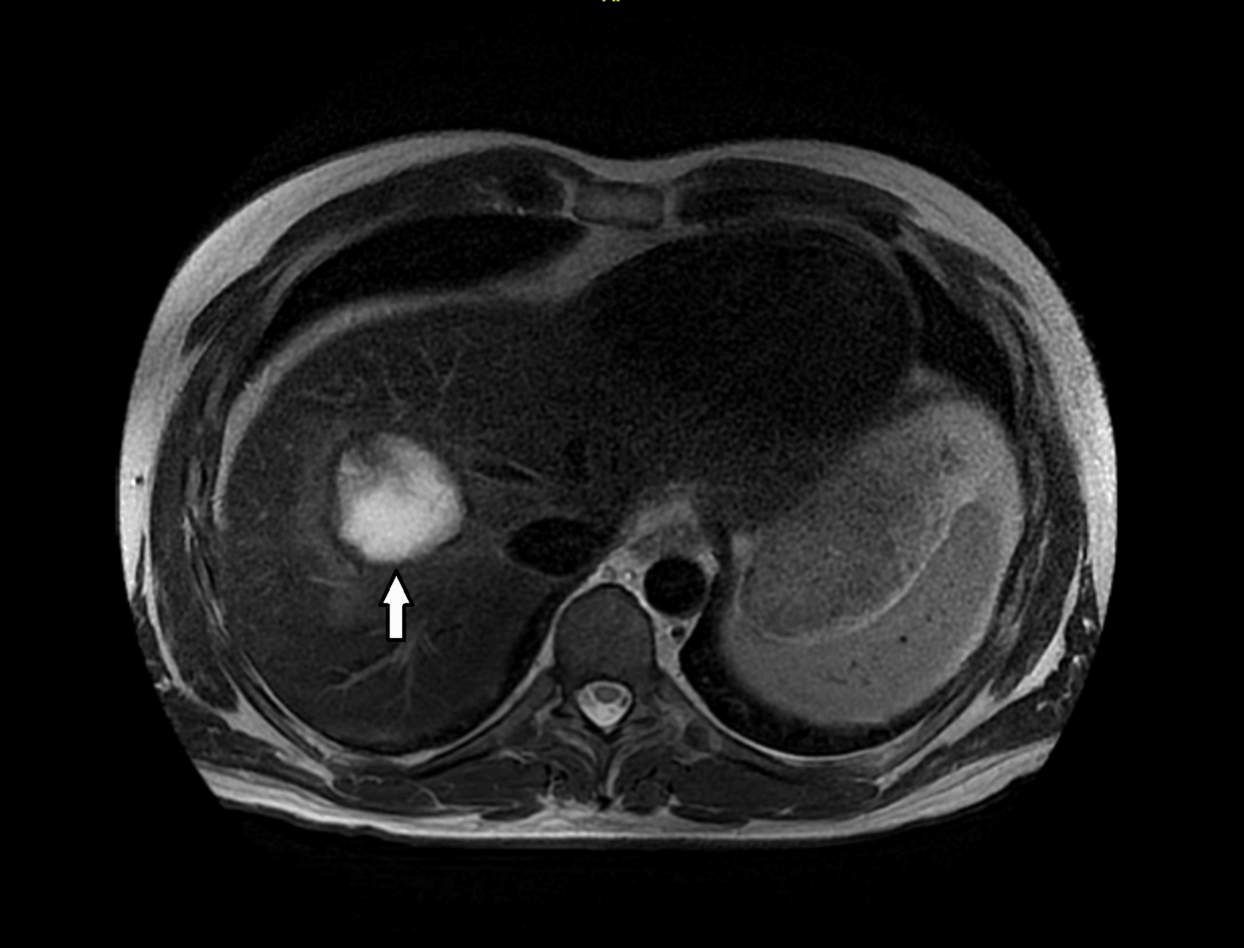
A magnetic resonance imaging scan with contrast of the abdomen. Post-ultrasound-guided drainage of the abscess showed reduction of the hepatic abscess measuring 3.2cm x 3.4cm (white arrow).

Due to the strong suspicion of a hydatid cyst, the rarity of the case, limited expertise in abscess drainage, and concerns about a potential anaphylactic reaction secondary to drainage, medical management was initially prioritized in hopes of achieving a favorable outcome. However, with no clinical improvement, the abscess was subsequently drained under ultrasound guidance by the interventional radiology team. Approximately 1708 ml of brownish, pus-filled fluid was drained and sent to the laboratory for analysis, including tests for ova, cysts, amoebic parasites, and hydatid serology and microscopy. All results came back negative. Post-drain imaging showed a reduction in size, and this was associated with significant improvement in clinical symptoms and subsiding spikes in fever (Figure 3).

Outcome and follow-up

After improvement in clinical symptoms, the patient was discharged with instructions for regular follow-up scans and outpatient gastroenterology reviews. A liver magnetic resonance scan conducted six months post-drainage revealed no significant change in the abscess compared to the immediate post-drain images. However, a follow-up magnetic resonance scan one year after the procedure showed a gradual resolution of the abscess. The patient continued to do well, maintaining a good appetite and overall health after treatment. Regular monitoring was recommended to ensure sustained progress, though no further complications were noted during the recovery period.

## Discussion

Liver abscesses are the most common type of visceral abscess, consisting of pus-filled lesions in the liver that may arise from liver injury or the spread of an intra-abdominal infection through the portal circulation. They are primarily categorized as pyogenic or amoebic, with a smaller percentage caused by parasites or fungi [1]. The annual incidence rate is around 2.3 cases per 100,000 individuals, with males being affected more frequently than females. Age also plays a role in the type of abscess, as people between 40 and 60 years old are more prone to developing non-traumatic liver abscesses [1]. Hydatid cyst is recognized by the World Health Organization as a neglected tropical disease [5]. The occurrence of a secondary bacterial infection in conjunction with a hydatid cyst is an extremely rare condition [6].

Hydatid disease, or echinococcosis, is caused by the larval stage of tapeworms from the genus *Echinococcus*, primarily* Echinococcus granulosus*. The adult tapeworm resides in the small intestine of carnivorous animals like dogs, foxes, and wolves, which are definitive hosts. These tapeworms release eggs in feces, which can be ingested by herbivorous and omnivorous animals, such as sheep, serving as intermediate hosts. Humans become infected through contact with dogs or by ingesting contaminated food, water, or soil. Once ingested, the eggs hatch in the intestine, releasing larvae that penetrate the intestinal wall and travel via the bloodstream, primarily settling in the liver [7]. Hydatid cysts in the liver can often remain asymptomatic for many years; however, they may eventually cause complications depending on their size, location, or infection. If a cyst becomes infected, it may mimic the symptoms of a liver abscess, leading to fever, chills, and weight loss [8].

Diagnosing these conditions can be challenging and relies on a combination of imaging studies and serological tests. Imaging techniques such as ultrasound, computed tomography, and magnetic resonance imaging are essential for identifying the distinctive features of hydatid cysts. Serological tests including enzyme-linked immunosorbent assay (ELISA), can confirm the diagnosis by detecting specific antibodies against Echinococcus [9].

For more than a decade, surgical treatment has been the only accepted approach for liver hydatid cysts. A stigma has persisted around surgical intervention, with the belief that hydatid disease presents an absolute contraindication for needle puncture or aspiration, as these procedures can significantly increase the risk of anaphylaxis, death, and the spread of the disease. Through experimentation, the careful aspiration of the hydatid cyst using the puncture, aspiration, injection, and re-aspiration (PAIR) technique has proven to be an effective management approach. Close monitoring for potential anaphylaxis is recommended postoperatively [10].

Praziquantel is known to be effective in treating hydatid cysts when administered at higher doses. It acts quickly and is particularly effective against protoscolices. Additionally, it serves as a good prophylactic agent, preventing the implantation of protoscolices and reducing the risk of recurrence. However, albendazole is considered more effective for treating the entire cyst, as praziquantel is less active against the germinal layer of the hydatid cyst [11].

## Conclusions

This case illustrates the complex clinical presentation of a young patient with a hydatid liver abscess complicated by bacterial superinfection. Initially, the diagnosis of a hydatid cyst was supported by clinical examination, travel history to an endemic region, and stool serological tests. However, the development of systemic symptoms prompted further investigation. Imaging studies confirmed a liver abscess, necessitating a dual treatment approach with antiparasitic medication and antibiotics to manage both the hydatid infection and the bacterial complication. This situation emphasizes the need for interdisciplinary management, with collaboration between medical and surgical teams to enhance patient outcomes. Prompt diagnosis and appropriate treatment are vital to prevent severe complications like abscess rupture or sepsis. Additionally, the case underscores the importance of considering alternative diagnoses in patients with atypical symptoms and the necessity of thorough clinical evaluations and targeted imaging to ensure timely and accurate diagnoses, ultimately improving patient care.

## References

[1] Akhondi H, Sabih DE (2023). Liver abscess. StatPearls.

[2] Derbel F, Mabrouk MB, Hamida MB (2012). Hydatid cysts of the liver - diagnosis, complications and treatment. Abdominal Surgery.

[3] Grosso G, Gruttadauria S, Biondi A, Marventano S, Mistretta A (2012). Worldwide epidemiology of liver hydatidosis including the Mediterranean area. World J Gastroenterol.

[4] Alexiou K, Mitsos S, Fotopoulos A (2012). Complications of hydatid cysts of the liver: spiral computed tomography findings. Gastroenterology Res.

[5] Castillo S, Manterola C, Grande L, Rojas C (2021). Infected hepatic echinococcosis. Clinical, therapeutic, and prognostic aspects. A systematic review. Ann Hepatol.

[6] García MB, Lledías JP, Pérez IG (2010). Primary super-infection of hydatid cyst--clinical setting and microbiology in 37 cases. Am J Trop Med Hyg.

[7] Kozielewicz DM, Sikorska K, Stalke P (2021). Liver abscesses - from diagnosis to treatment. Clin Exp Hepatol.

[8] Geiger LE (1965). Hydatid cyst of the brain. Report of a case. J Neurosurg.

[9] Eckert J, Deplazes P (2004). Biological, epidemiological, and clinical aspects of echinococcosis, a zoonosis of increasing concern. Clin Microbiol Rev.

[10] Khuroo MS (2021). Percutaneous drainage in hepatic hydatidosis-the PAIR technique: concept, technique, and results. J Clin Exp Hepatol.

[11] Arif SH, Malik AA, Khaja AR, Dass TA, Naikoo ZA (2011). Role of albendazole in the management of hydatid cyst liver. Saudi J Gastroenterol.

